# Identification of miRNAs Affecting the Establishment of *Brassica Alboglabra* Seedling

**DOI:** 10.3389/fpls.2016.01760

**Published:** 2016-11-22

**Authors:** Rongfang Guo, Yanping Deng, Zhongkai Huang, Xiaodong Chen, Xu XuHan, Zhongxiong Lai

**Affiliations:** ^1^College of Horticulture, Fujian Agriculture and Forestry UniversityFuzhou, China; ^2^Institute of Horticultural Biotechnology, Fujian Agriculture and Forestry UniversityFuzhou, China; ^3^Institut de la Recherche Interdisciplinaire de ToulouseToulouse, France

**Keywords:** seedling establishment, miRNA, 24 nt, Chinese kale, *Brassica alboglabra*

## Abstract

MicroRNAs (miRNAs) are important for plant development including seed formation, dormancy, and germination, as well as seedling establishment. The *Brassica* vegetable seedling establishment stage influences the development of high quality seedlings, but also affects the nutrient content of sprouts. Chinese kale (*Brassica alboglabra*) seedlings at different growth stages were used to construct two small-RNA (sRNA) libraries. We comprehensively analyzed the miRNAs in 2- and 9-day-old seedlings. An average of 11,722,490 clean reads were generated after removing low-quality reads and adapter contaminants. The results revealed that 37.65 and 26.69% of the sRNAs in 2- and 9-day-old seedlings, respectively, were 24 nt long. In total, 254 known mature miRNA sequences from 228 miRNA families and 343 novel miRNAs were identified. Of these miRNAs, 224 were differentially expressed between the two analyzed libraries. The most abundant miRNAs identified by sequence homology were miR156, miR167, and miR157, each with more than 100,000 sequenced reads. Compared with the expression levels in 2-day-old seedlings, *MiR8154* and *miR390* were the most up- and down-regulated miRNAs respectively in 9-day-old seedlings. Gene ontology enrichment analysis of the differentially expressed-miRNA target genes affecting biological processes revealed that most genes were in the “regulation of transcription” category. Additionally, the expression patterns of some miRNAs and target genes were validated by quantitative real-time polymerase chain reaction. We determined that development-associated miRNAs (e.g., *bal-miR156/157/159/166/167/172/396*), were highly-expressed during seedling-establishment stage, as were stress-related (*bal-miR408*) and metabolism-related (*bal-miR826*) miRNAs. Combined with the low level of targets *SPL9* and *AP2*, it was concluded that *miR156-SPL9* and *miR172-AP* modules play key roles during the *B. alboglabra* seedling establishment stage.

## Introduction

Germination involves seed metabolic activities which result in the emergence of the radicle and cotyledons, and eventually the establishment of a seedling. The appearance of the radicle marks the end of germination and the initiation of the seedling stage. Plant growth during the seedling-establishment stage is nutritionally dependent on the mobilization of reserves until leaves develop and initiate photosynthesis (Nonogaki, 2010; Bewley et al., 2012). Chinese kale (*Brassica alboglabra* Bailey) is a cruciferous biennial vegetable crop originated in southern China (Sun et al., 2011a,b, 2012; Wei et al., 2011). Sprouts of Chinese kale are consumed lately as they contain an abundance of functional substances especially glucosinolates. After 9 days of cultivation, the first true leaf of *B. alboglabra* emerges and the seedling enters the next vegetative growth stage. As the seedling develops, its glucosinolate contents are decreased.

Plant microRNAs (miRNAs) are short (21–24 nt) non-coding molecules that regulate gene expression by influencing the mRNA cleavage or the inhibition of protein translation (Llave et al., 2002; Bartel, 2004; Brodersen et al., 2008). Mutant seedlings with an excessive accumulation of miRNA-resistant transcripts and transgenic plants overexpressing a specific miRNA have been analyzed in several genomic studies to elucidate the functions of plant miRNAs (Linsen et al., 2009; Liu et al., 2014; Lu et al., 2014; Gupta and Nath, 2015; Jia et al., 2015). Multiple developmental factors such as the timing and patterns of organ formation (Mallory et al., 2005; Wang et al., 2005; Wollmann et al., 2010; Rubio-Somoza and Weigel, 2011), which are under the control of the miRNA genes and miRNA targets, have been identified and characterized. The functions of plant miR156, miR159, miR160, miR167, miR172, miR390, and miR402 during seed development, dormancy, and germination have been verified in *Arabidopsis thaliana* and *Brassica napus* (Liu et al., 2007; Reyes and Chua, 2007; Kim et al., 2010; Martin et al., 2010a,b; Huang et al., 2013). Investigations of miRNAs in the seeds of *Brassica* vegetable have mainly focused on identifying miRNAs in *B. napus* seeds. The abundance of miRNAs differs between developing and mature *B. napus* seeds, and the predicted target genes are mostly related to development, energy metabolism and storage (Körbes et al., 2012; Zhao et al., 2012; Huang et al., 2013). *Brassica napus* seeds with differing oil contents also exhibit variable gene expression levels (Zhao et al., 2012). The miRNA and transcription factors or other target genes are expressed during embryogenesis, seed maturation, imbibition, and seedling establishment, suggesting that the down-regulation of target genes by miRNA may be critical for seed formation, germination, and seedling development (Pluskota et al., 2011). However, only a few miRNAs and their target genes associated with the seedling-establishment stage have been analyzed.

The proper establishment of *Brassica* vegetable seedlings influences the development of high-quality mature seedlings that are used as food sprout in diet but it also affects the nutrient content of the sprout. To systematically identify miRNAs and other small RNAs (sRNA) potentially involved in regulating *B. alboglabra* seedling establishment, we constructed sRNA libraries from 2- and 9-day-old seedlings and profiled the sRNA expression levels by means of the Illumina/Solexa sequencing technology. Targets of differentially expressed miRNAs were predicted using the psRobot and TargetFinder programs. To further investigate the role of miRNAs during the establishment of Chinese kale seedlings, we analyzed the expression patterns of miRNA and their targets in the cotyledons were detected using quantitative real-time polymerase chain reaction (qRT-PCR).

## Materials and methods

### Plant material

Seeds of Chinese kale (*Brassica alboglabra*) were immersed in 0.7% sodium hypochlorite for 30 min, then drained and washed with distilled water until they reached neutral pH value. They were then placed in distilled water and soaked overnight. The seeds were grown in petri dishes with wet filter papers. All of the seeds and seedlings were grown under a 16-h light and 8-h dark photoperiod and a constant 25°C temperature in an incubator (150 μ E m ^−2^ s ^−1^). The radicles grew out from the seed coats and rapidly elongated at the 2nd day, thus a seedling was forming, a stage called seedling-establishment stage. The establishment was finished until the 9th day when the true leaf emerged. Finally, seedlings after soaked for 0–10 days were collected for measurements. The seed and seedling samples were rapidly and gently collected from the surface of the filter paper, then were frozen in liquid nitrogen immediately and kept in polyethylene bags at −80°C for RNA extraction and glucosinolates analysis. In this study, two sRNA libraries were generated with 2- and 9-day-old Chinese kale seedlings. These two libraries comprised all of the miRNAs during the establishment of Chinese kale seedlings.

### Measurement of glucosinolate content

Glucosinolates were extracted and analyzed as previously described with minor modifications (Guo et al., 2011). Samples (500 mg) were boiled in 3 mL water for 10 min. After transferring the supernatant to a new tube, the residues were washed with water (3 mL), and the combined aqueous extract was applied to a DEAE-Sephadex A-25 (30 mg) column (pyridine acetate form) (Sigma, St. Louis, MO, USA). The column was washed three times with 20 mM pyridine acetate and twice with water. The glucosinolates were converted into their desulfo analogs by overnight treatment with 100 μL of 0.1% (1.4 units) aryl sulfatase (Sigma, St. Louis, MO, USA) added into the column, and the desulfoglucosinolates were collected by eluting with 2 × 0.5 mL water. HPLC analysis was performed using an HPLC system consisting of an Agilent HPLC series chromatograph (Agilent Technologies). A Hypersil C18 column (5 μm particle size, 4.6 mm × 250 mm; Elite Analytical Instruments Co., Ltd., Dalian, China) was used with a mobile phase of acetonitrile and water at a flow rate of 1.0 mL/min. The procedure was 1.5% acetonitrile for 0–5 min, then a linear gradient to 20% acetonitrile over the next 15 min followed by isocratic elution with 20% acetonitrile for the final 10 min. A 40 μL sample was autosampled into the column. The desulfoglucosinolates were detected at 226 nm with ortho-nitrophenyl-β-d-galactopyranoside (Sigma, St. Louis, MO, USA) as an internal standard. The glucosinolate content was presented as μmol/g dry weight (dw).

### Construction of *B. alboglabra* small RNA libraries and solexa sequencing

Total RNA was isolated from the samples mentioned above using TRIzol Reagent kit (Invitrogen, Life Technologies, Carlsbad, CA) according to the manufacturer's instructions, and pooled in an equal fraction ratio for the construction of a Chinese kale small RNA library (SRP076430, SRS1497183, SRX1837757). After that the small RNA fragments of 16–30 nt were isolated from a 15% PAGE gel and purified, and then the small RNAs were ligated to a 5′ RNA adapter (5′-GUUCAGAGUUCUACAGUCCGACGAUC-3′) and a 3′RNA adapter (5′-pUCGUAUGCCGUCUUCUGCUUGidT-3′; p, phosphate; idT, inverted deoxythymidine) sequentially using T4 RNA ligase. The samples were reversely transcribed to cDNA with the RT primer (5′-CAAGCAGAAGACGGCATACGA-3′) using Superscript II reverse transcriptase (Invitrogen), and amplified by PCR. Finally, the small RNAs from samples were sequenced using Solexa sequencing technology.

### Analysis of small RNA of *B. alboglabra*

In order to predict the potential conserved and novel miRNAs of *B. alboglabra*, bioinformatics analysis of the Solexa sequencing data was conducted. Firstly, after cleaning the 35 nt sequence tags and removing several kinds of contaminants and low quality tags, the length distribution of clean tags was summarized. Secondly, the clean tags were compared against non-coding RNAs from Rfam database (http://www.sanger.ac.uk/software/Rfam) and the NCBI GenBank database (http://www.ncbi.nlm.nih.gov/) to classify the degradation fragments of non-coding RNAs. The unique sRNA sequences were used to search for miRNA sequences using miRBase 21 to identify the conserved miRNAs in Chinese kale. Lastly, the novel miRNAs from the surplus unannotated small RNAs were predicted by Mireap. Analyzed sRNAs are only considered candidate miRNA genes if they fulfill the following criteria: (1) a mature sequence localized in one arm of the stem-loop structure and between 19 and 24 nt; (2) the corresponding miRNA^*^ sequence identified; (3) the pre-miRNA sequence folded into an appropriate stem-loop hairpin secondary structure; (4) the mfe of secondary structures ≤ −20 kcal/mol; and (5) no more than 7-nt mismatches in the miRNA:miRNA^*^ duplex (Meyers et al., 2008).

### Prediction of targets of novel miRNAs in *B. alboglabra*

It is necessary to identify and characterize the targets of novel miRNAs in order to understand their biological functions. In the present experiment, the potential targets of Chinese kale novel miRNAs were predicted using the psRNATarget program (http://plantgrn.noble.org/psRNATarget/). Newly identified kale novel novel miRNAs sequences were used as custom miRNA sequences and citrus EST and nucleotide databases were used as custom plant databases. The rules used for Chinese kale novel miRNAs targets prediction are based on those suggested by Allen et al. (2005) and Schwab et al. (2005): (1) No more than four mismatches between sRNA and target (G-U bases count as 0.5 mismatches); (2) No more than two adjacent mismatches in the miRNA/target duplex; (3) No adjacent mismatches in positions 2–12 of the miRNA/target duplex (5′ of miRNA); (4) No mismatches in positions 10–11 of miRNA/target duplex; (5) No more than 2.5 mismatches in positions 1–12 of the miRNA/target duplex (5′ of miRNA); (6) Minimum free energy (MFE) of the miRNA/target duplex should be ≥75% of the MFE of the miRNA bound to its perfect complement.

### Real-time quantitative PCR of miRNAs and their targets in *B. alboglabra*

The qPCR was used to validate results obtained from high throughput sequencing of Chinese kale miRNAs and their targets. RNA samples were reverse transcribed using an One Step PrimeScript miRNA cDNA Synthesis Kit (Perfect Real Time) (Takara Code: D350A) and PrimeScript® RT reagent Kit (Takara Code: DRR037A) for miRNA and target genes tested respectively. Expression profiles of 12 miRNAs and 10 targets were examined using an SYBR® PrimeScript™ miRNA RT-PCR Kit (Takara Code: RR716) and SYBR® Premix Ex Taq™ II (Tli RNaseH Plus) (Takara Code: RR820A) respectively. All reactions were performed in triplicate in a LightCycler 480 qPCR instrument (Roche Applied Science, Switzerland), with a dissociation curve used to control for primer dimers in the reactions. *Actin 2* (Bol022870 locus=C03:23167671:23168969:+) was used as endogenous control for quantification. Expression of genes from 1-day-old seedlings was set to 1.0 as calibrator. The primer sequences of miRNA and target genes are provided in Supplementary Table S7.

### Statistical analysis

**Figure 1:**
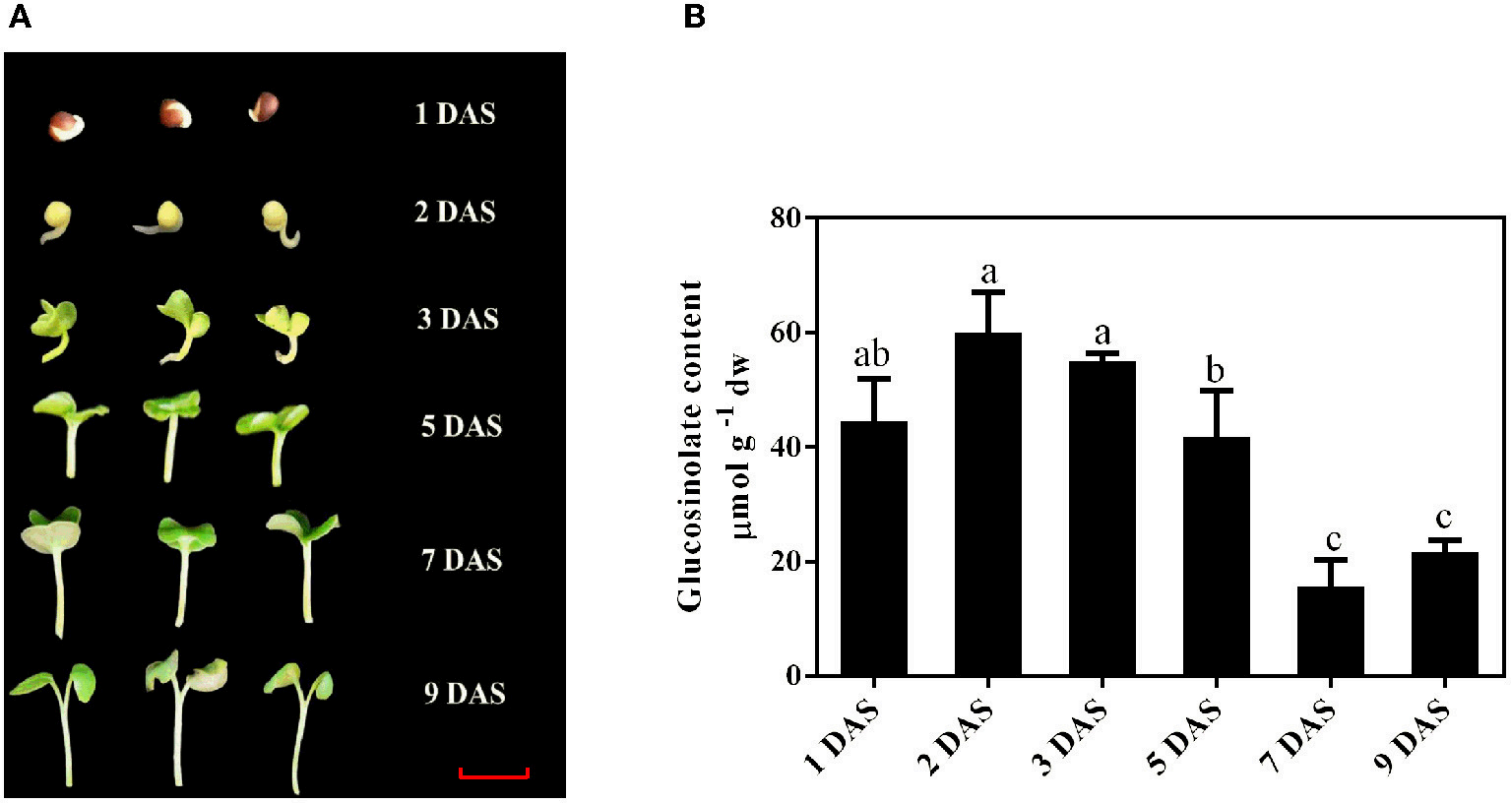
**Establishment of *B. alboglabra* seedlings and determinations of their glucosinolate content. (A)** Establishment of *B. alboglabra* seedlings. DAS, day after soaking; 1 DAS, seed imbibition; 2 DAS, emergence of radical (germinated seed, the initiation of seedling establishment); 3 DAS, appearance of cotyledon; 5 DAS: seedling elongation; 7 DAS, seedling elongation; 9 DAS, seedling establishment. The scale bar represents for 1 centimeter. **(B)** The content of glucosinolates in *B. alboglabra* seedlings at different growth stages. The differences labeled with different letters were considered significant at *p* < 0.05.

Statistical analysis was performed using the SPSS package program version 11.5 (SPSS, Chicago, IL, USA). The data were analyzed by one-way ANOVA followed by Tukey's *post-hoc* test and differences labeled with different letters were considered significant at *p* < 0.05. The values are reported as means ± standard error (SE) for Figures 1, **6**, **8**.

## Results

### Establishment of *B. alboglabra* seedlings and determinations of their glucosinolate content

The establishment of *B. alboglabra* seedlings was showed in Figure 1A. Germination commences with the uptake of water by the dry seed imbibition and is completed when the radicle penetrates the structures that surround it 1 day after soaking (DAS). This signified marking the end of germination and the initiation of the seedling-establishment stage. Then the hypocotyl began to elongate 2 DAS and cotyledon appeared 3 DAS. The establishment was finished at 9 DAS when the true leaf emerged. As the radicle and cotyledons grew, the glucosinolate content decreased. After 9 days, the glucosinolate content of seedlings was 21.65 μmol g ^−1^ dw, which was only 36.09% of that in 2-day-old seedlings (Figure 1B).

### Deep sequencing of *B. alboglabra* small RNAs

To identify miRNAs involved in the establishment of Chinese kale seedlings, two sRNA libraries were generated from the shoots of 2- and 9-day-old seedlings and sequenced using an Illumina/Solexa analyzer. For 2-day-old seedlings, 11,790,430 raw reads were generated, including 96,630 low-quality reads and 11,693,800 high-quality reads. In contrast, for the 9-day-old seedlings, 12,014,795 raw reads were generated, including 96,309 low-quality reads and 11,918,486 high-quality reads. After removing the low-quality reads and adapter contaminants, 11,605,016 and 11,839,963 clean reads were obtained for the 2- and 9-day-old seedlings, respectively. The clean reads included 4,883,843 and 4,144,593 unique sequences for the 2- and 9-day-old seedlings, respectively, with lengths of 18–30 nt (Table 1). According to the general principle of miRNA analysis, the sRNAs were compared with the *Brassica oleracea* reference genome (http://brassicadb.org/brad/index.php) to identify matching sequences. Based on the results of a short oligonucleotide analysis package (SOAP), 4,818,544 (41.52%) sequences from 2-day-old seedlings were identical to genome sequences, including 1,396,200 (28.59%) unique sequences. For the sequences from 9-day-old seedlings, 5,263,854 (44.46%) exactly matched genome sequences, including 1,212,378 (29.25%) unique sequences.

**Table 1:** **The summary of high-throughput sequencing data of *B. alboglabra* small RNA libraries that constructed with 2- and 9-day-old seedlings**.

**Category**	**2-day-old seedling**		**9-day-old seedling**	
	**Reads No**.	**Percentage**	**Reads No**.	**Percentage**
Total reads	11,790,430		12,014,795	
High quality reads	11,693,800	100	11,918,486	100
Clean reads	11,605,016	99.24	11,839,963	99.34
Total sRNAs	11,605,016	100	11,839,963	100
Total sRNAs mapping to genome	4,818,544	41.52	5,263,854	44.46
Unique sRNAs	4,883,843	100	4,144,593	100
Unique sRNAs mapping to genome	1,396,200	28.59	1,212,378	29.25

A comparison against all plant miRNA precursors and mature miRNAs in miRBase 21 (http://www.mirbase.org/) revealed that among the unique sequences of 2- and 9-day-old seedlings, 30,322 (0.62%) and 28,292 (0.68%) were similar to known miRNAs, respectively (Kozomara and Griffiths-Jones, 2014). Other unique sequences, including ribosomal RNAs (2-day-old seedlings: 64,038, 1.31%; 9-day-old seedlings: 67,188, 1.62%), small nuclear RNAs (3,185, 0.07%; 2780, 0.07%), small nucleolar RNAs (411,090, 8.42%; 340,626, 8.22%), and transfer RNAs (11,543, 0.24%; 12,218, 0.29%), were also identified following a BLASTN search of the Rfam (11.0) database (http://rfam.janelia.org/) (Nawrocki et al., 2014; Table 2).

**Table 2:** **Types of small RNA (sRNA) detected in 2- and 9-day-old *B. alboglabra* seedlings**.

**Type**	**Unique sRNA**	**Percentage**	**Total sRNA**	**Percentage**
Exon antisense	26,369/31,013	0.54/0.75	58,389/75,047	0.5/0.63
Exon sense	49,888/46,991	1.02/1.13	105,559/106,165	0.91/0.90
Intron antisense	44,263/39,890	0.91/0.96	136,659/138,386	1.18/1.17
Intron sense	62,353/59,265	1.28/1.43	236,331/235,827	2.04/1.99
miRNA	30,322/28,292	0.62/0.68	1,589,413/2,074,251	13.7/17.52
rRNA	64,038/67,188	1.31/1.62	735,479/878,767	6.34/7.42
snRNA	3,185/2,780	0.07/0.07	6,769/5,434	0.06/0.05
snoRNA	411090/340,626	8.42/8.22	710,687/673,971	6.12/5.69
tRNA	11,543/12,218	0.24/0.29	157,864/429,629	1.36/3.63
Unannotated reads	4,180,792/3,516,330	85.6/84.8	7,867,866/722,2486	67.8/61

*The former data is the number or percentage of sRNA in 2-day-old seedlings. The latter is the number or percentage of sRNA in 9-day-seedlings*.

**Figure 2:**
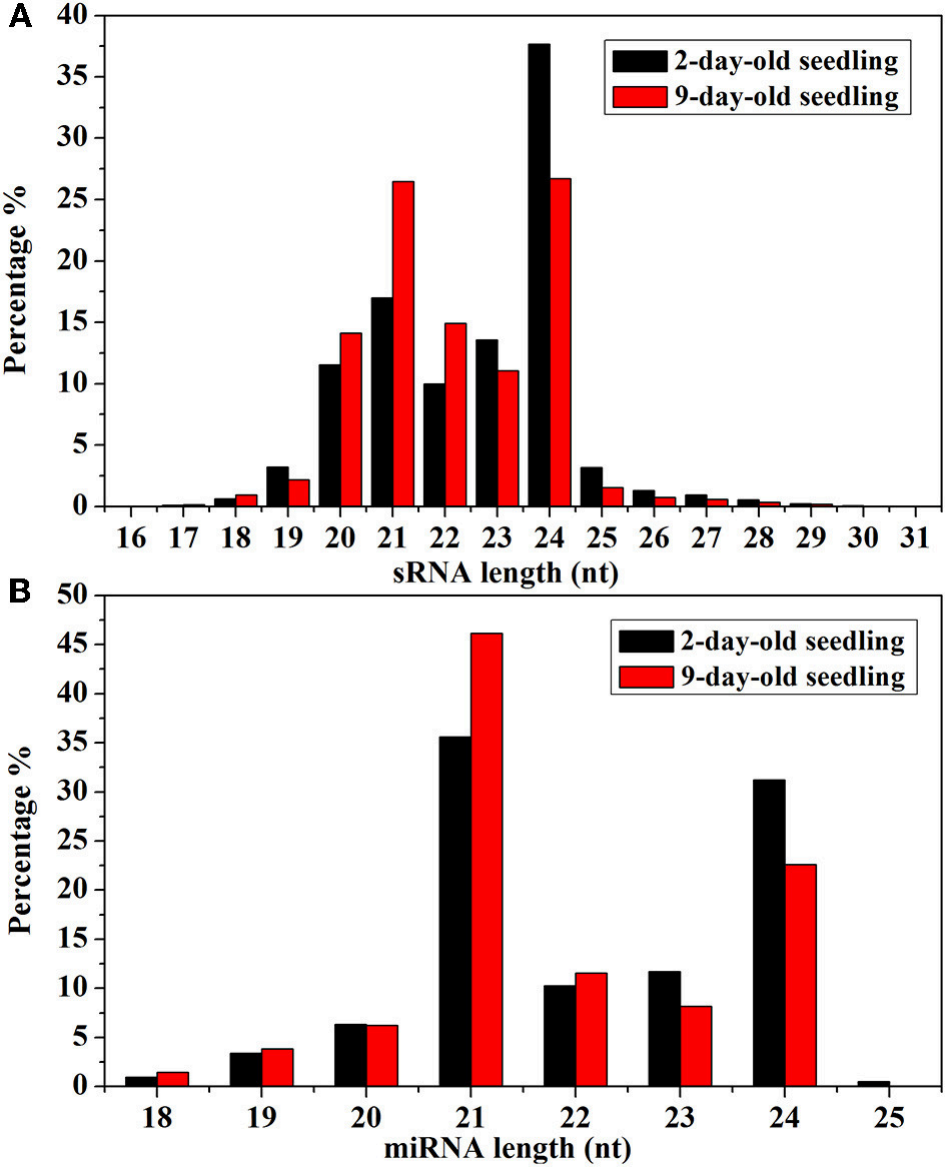
**(A)** Size distributions of sRNA in 2- and 9-day-old *B. alboglabra* seedlings. The X axis represented the lengths of sRNA ranging from 16 to 31 nucleotides. The Y axis is the percentage of sRNA at a specific length. Black column: size distribution of sRNA in 2-day-old seedlings; Red column: size distribution of sRNA in 9-day-old seedlings. **(B)** Size distributions of miRNA in 2- and 9-day-old *B. alboglabra* seedlings. The X axis represented the lengths of miRNA ranging from 18 to 25 nucleotides. The Y axis is the percentage of miRNA numbers at a specific length. Black column: size distribution of miRNA in 2-day-old seedlings; Red column: size distribution of miRNA in 9-day-old seedlings.

Analyses of the unique Chinese kale sRNA sequences revealed an uneven size distribution pattern (Figure 2). Most unique sRNAs were 20–24 nt long. Of the unique sRNAs from 2-day-old seedlings, most consisted of 24 nt (37.65%), 21 nt (17.00%), or 23 nt (13.58%). In contrast, the majority of the unique sRNAs from 9-day-old seedlings consisted of 24 nt (26.69%), 21 nt (26.47%), or 22 nt (14.92%). A similar trend was also observed for the miRNA size distributions (i.e., decrease in the percentage of 24-nt miRNAs over time).

### Identification of *B. alboglabra* conserved miRNAs

To identify conserved *B. alboglabra* miRNAs, the generated libraries were compared with the complete set of mature plant miRNA sequences available in miRBase 21 (http://www.mirbase.org/ftp.shtml). In total, 254 known mature miRNA sequences belonging to 228 plant miRNA families were identified. Of these sequences, 216 were identified in the 2-day-old seedling library, 218 were identified in the 9-day-old seedling library, and 180 were detected in both libraries. With three members each, the most commonly detected families were miR156, miR390, and miR396. Additionally, 21 families, including miR157, miR158, and miR168 contributed two members. However, most miRNA families were represented by a single member (Supplementary Table S1).

**Figure 3:**
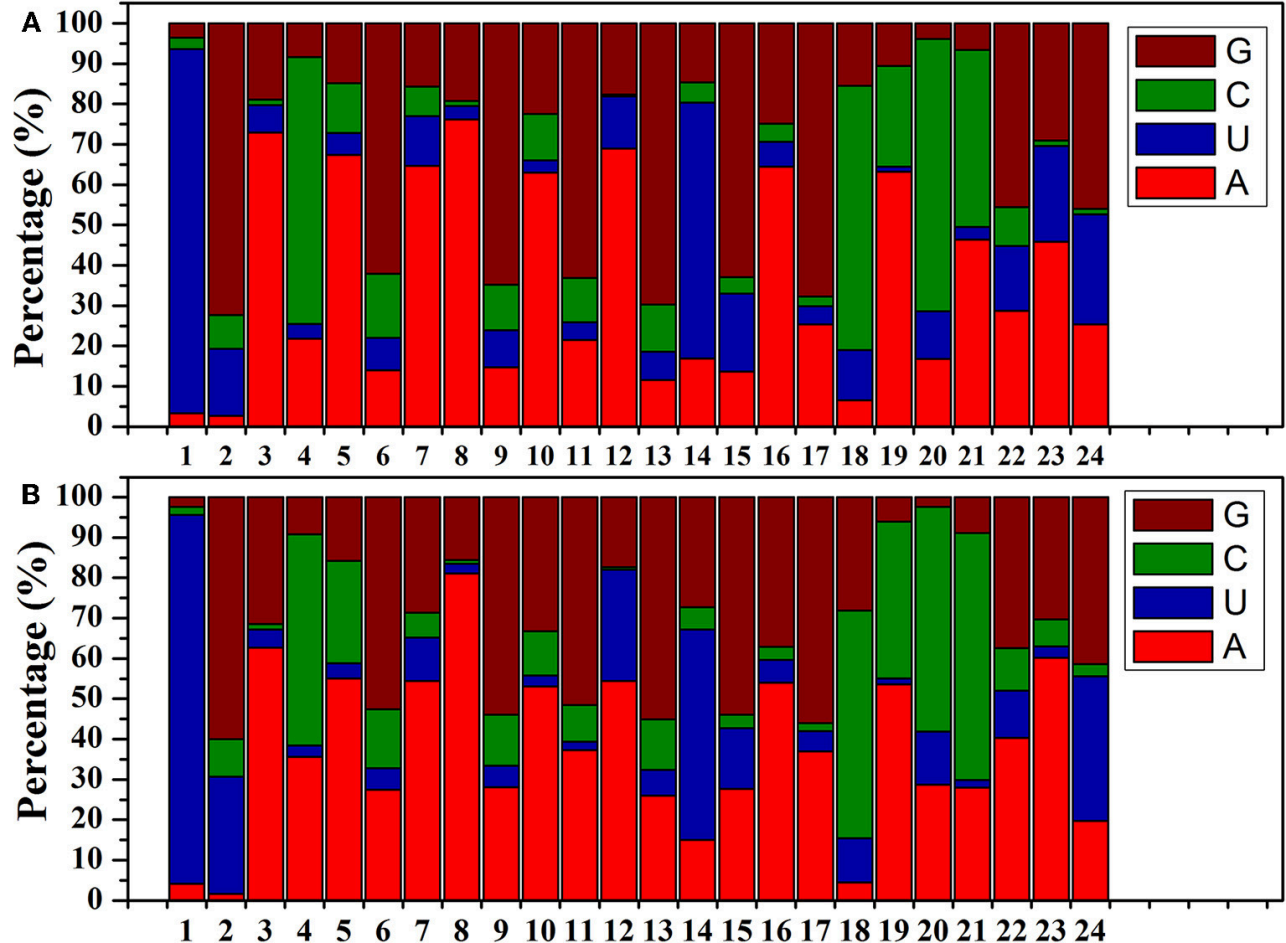
**(A)** miRNA nucleotide bias at each position in 2-day-old *B. alboglabra* seedlings. **(B)** miRNA nucleotide bias at each position in 9-day-old *B. alboglabra* seedlings. The X axis represented the position of miRNA nucleotide. The Y axis is the percentage of a specific nucleotide numbers at each position of miRNA nucleotide. Brown column: Guanine (G); Green column: Cytosine (C); Blue column: Uracil (U); Light red column: Adenosine (A).

The size distributions (18–25 nt) of the known *B. alboglabra* miRNAs are provided in Figure 2B. Most of the miRNAs were 21 nt (2-day-old seedlings: 35.61%; 9-day-old seedlings: 46.15%) or 24 nt (31.22%; 22.60%) long. Most of the miRNA sequences in the *B. alboglabra* libraries started with a uridine (Figure 3), which was consistent with the results of previous studies involving plant miRNA sequences (Czech and Hannon, 2011).

The number of reads of known *B. alboglabra* miRNAs ranged from 5 to 999,511. Most of the sequenced miRNAs (80.71%) were represented by fewer than 1000 reads. Generally, the 216 known miRNAs were classified into two categories, with conserved miRNAs expressed more than non-conserved miRNAs. The miR156 sequence was the most abundant, with 932,225 and 999,511 reads detected in 2- and 9-day-old seedling libraries respectively, followed by miR167 and miR157 (Table 3).

**Table 3:** **Reads and sequence of 10 known *B. alboglabra* miRNAs**.

**miRNA name**	**Reads in 2-day-old seedling**	**Reads in 9-day-old seedling**	**Sequence**	**Length (nt)**	**5′/3′**
miR156a	932225	999511	TGACAGAAGAGAGTGAGCAC	20	5′
miR167a	161063	191301	TGAAGCTGCCAGCATGATCTA	21	5′
miR157a	126532	465483	TTGACAGAAGATAGAGAGCAC	21	5′
miR166a	51639	76928	TCGGACCAGGCTTCATTCCCC	21	3′
miR168a	50496	36304	TCGCTTGGTGCAGGTCGGGAC	21	–
miR158	38333	25912	TTTCCAAATGTAGACAAAGCA	21	3′
miR158	29838	23776	CTTTGTCTATCGTTTGGAAAAG	22	5′
miR3946	16420	28171	TTGAGAGAAGAGAGCGAGCAC	21	–
miR2118	13182	11608	GTCGATGGAACAATGTAGGCAAGG	24	5′
miR8167a	12582	11531	GGAGATGGTGGAGATGGTGGGGAT	24	–

### Prediction of novel *B. alboglabra* miRNAs

To identify novel miRNAs, 4,180,792 and 3,516,330 un-annotated unique sequences from 2- to 9-day-old seedlings, respectively, were mapped to the *B. oleracea* genome. The sequences that were identical to genome sequences were analyzed to predict the secondary structure of each locus using the MIREAP program. Novel miRNA candidates were selected based on the secondary structure of precursor sequences, the miRNA/miRNA^*^ duplex, and the minimum free energy value. The results revealed that 343 sRNA sequences met the criteria for designating miRNAs as novel (Supplementary Table S2). Most of the novel miRNAs (73.69%) were 21 nt long and no novel 24 nt miRNAs were found.

**Figure 4:**
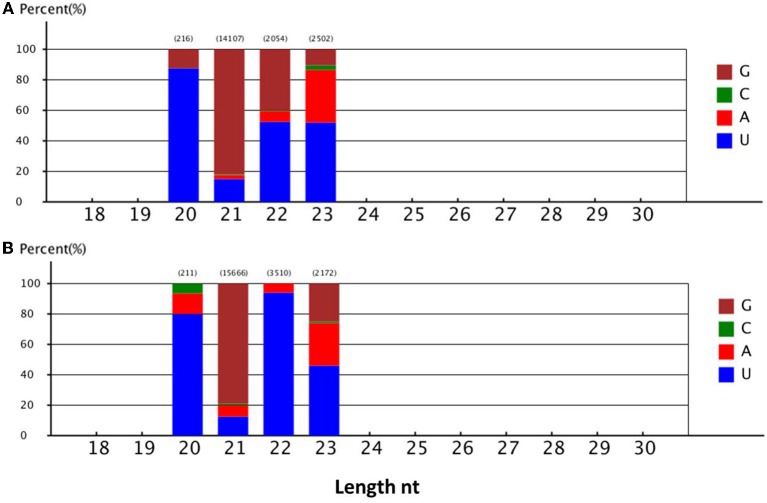
**(A)** First nucleotide bias of the different lengths' novel miRNA in 2-day-old *B. alboglabra* seedlings. **(B)** First nucleotide bias of the different lengths' novel miRNA in 9-day-old *B. alboglabra* seedlings. The X axis represented the length of novel miRNA. The Y axis is the percent of the first nucleotide numbers at each length of novel miRNA. Brown column: Guanine (G); Green column: Cytosine (C); Blue column: Uracil (U); Light red column: Adenine (A).

The number of novel *B. alboglabra* miRNA reads varied from 5 to 6057. Of these sequences, *Bal_novel_mir177* was the most highly expressed, followed by *Bal_novel_mir144* and *Bal_novel_mir94*. Only 7.34% of the novel miRNAs were expressed over 100 times (Supplementary Table S2). Analyses of the first nucleotide of the novel miRNAs indicated that more than 45% of the miRNAs consisting of 20, 22, or 23 nt began with a uridine, while only 13.56% of the miRNAs with 21 nt started with this nucleotide (Figure 4).

### miRNAs expression patterns during *B. alboglabra* seedling establishment

**Figure 5:**
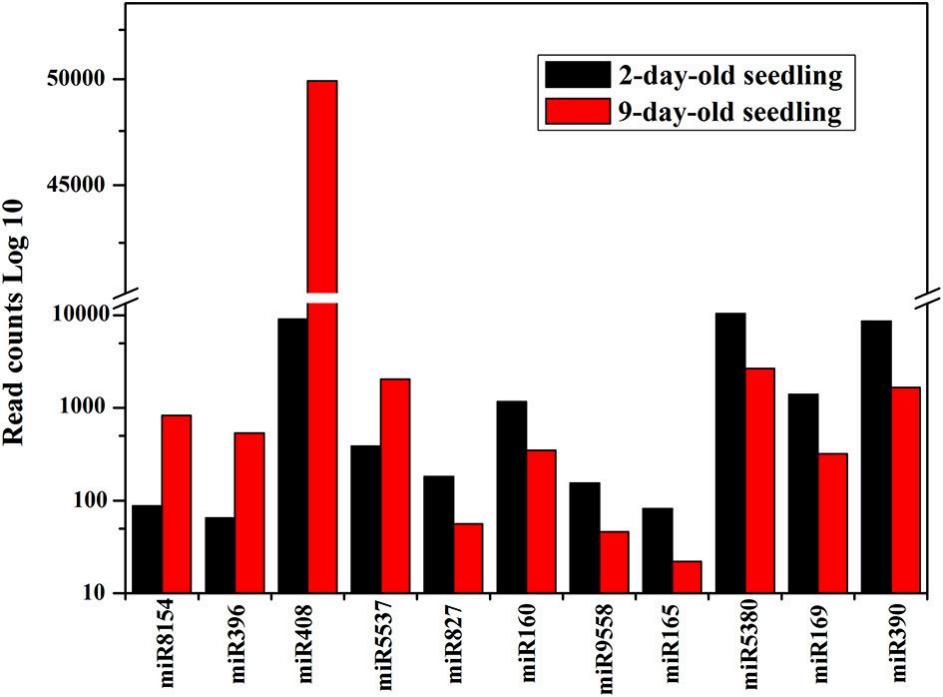
**Read counts of differentially represented known miRNAs which exhibited more than 2-fold number changes in 2- and 9-day-old seedling libraries**. The X axis represented miRNAs with more than 2-fold number changes in 2- and 9-day-old seedling libraries. The Y axis is the read counts (Log 10) of differentially represented miRNAs in 2- and 9-day-old seedling libraries. Black column: read counts of miRNA in 2-day-old seedlings; Red column: read counts of miRNA in 9-day-old seedlings.

The most abundant miRNAs in the analyzed libraries were miR156, miR167, and miR157, each with more than 100,000 sequenced reads. Comparisons of the normalized data suggested that 224 known and 181 novel miRNAs were differentially expressed between the two libraries. These miRNAs were classified into four categories. One group consisted of miRNAs expressed only in 2-day-old seedlings (50 known and 92 novel miRNAs). Of the 50 known miRNAs, only three were conserved miRNAs (i.e., miR171b-5p, miR156e-3p, and miR396a-5p). Another group comprised miRNAs expressed only in 9-day-old seedlings (56 known and 79 novel miRNAs). Among the 56 known miRNAs, six miRNAs (10.71%; i.e., miR319, miR394-3p, miR396b-5p, miR172e-3p, miR156f-3p, and miR171b) were conserved. A third group included miRNAs up-regulated in 9-day-old seedlings (41 known and six novel miRNAs). The fourth group consisted of miRNAs down-regulated in 9-day-old seedlings (77 known and four novel miRNAs) (Supplementary Tables S3, S4). The large number of sequences generated by high-throughput sequencing enabled the use of read counts in libraries to estimate miRNA abundance (Linsen et al., 2009). The differentially expressed known miRNAs that exhibited more than 2-fold number changes in the 2- and 9-day-old seedling libraries are presented in Figure 5. The most up- and down-regulated miRNAs in 9-day-old seedlings were *miR8154* and *miR390*, respectively. Additionally, miRNAs from the same family exhibited different expression patterns. For example, miR156a-5p was the most abundant miRNA in both libraries, while miR156f-3p was not detected in 2-day-old seedlings and miR156e-5p was not expressed in 9-day-old seedling. A similar situation was observed for the family MIR396 and MIR390 (Supplementary Table S3).

**Figure 6:**
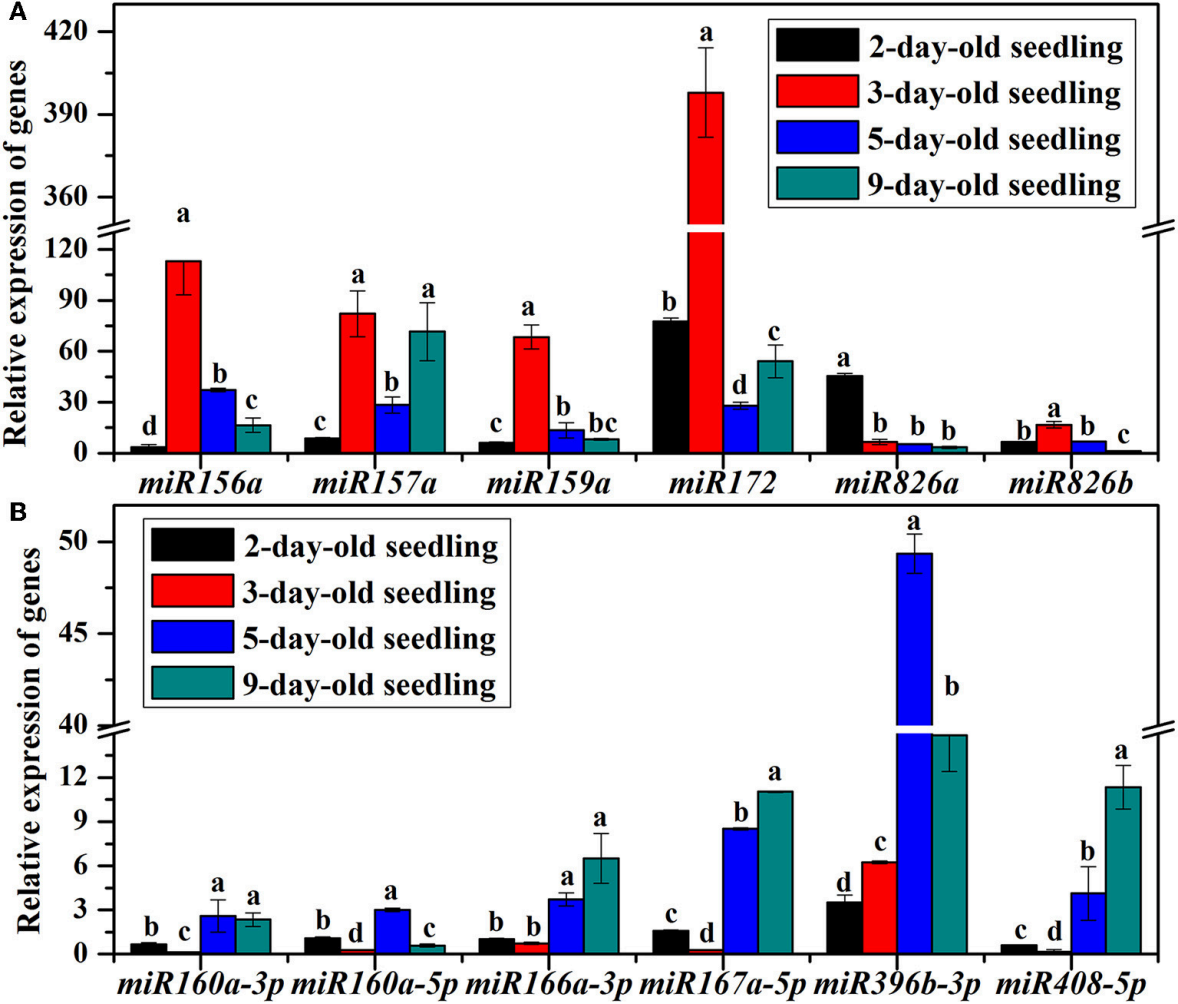
**(A)** Relative expression of miR156a, miR157a, miR159a, miR172, miR826a, and miR826b in 2, 3, 5, and 9-day-old *B. alboglabra* seedlings. **(B)** Relative expression of miR160a-3p, miR160a-5p, miR166a-3p, miR167a-5p, miR396b-3p, and miR408-5p in 2-, 3, 5, and 9-day-old *B. alboglabra* seedlings. The X axis represented miRNAs analyzed in *B. alboglabra* The Y axis is the relative expression of miRNAs. Black column: relative expression of miRNA in 2-day-old seedlings; Red column: relative expression of miRNA in 3-day-old seedlings; Blue column: relative expression of miRNA in 5-day-old seedlings; Indigo column: relative expression of miRNA in 9-day-old seedlings. The expressions of miRNAs were calculated relative to those in 1-day-old seedlings. Error bars indicate the SE of three replicates. The differences labeled with different letters were considered significant at *p* < 0.05.

To clarify the role of miRNAs during the *B. alboglabra* seedling-establishment stage, the expression of 12 miRNAs in 1-, 2-, 3-, 5-, and 9-day-old seedlings were analyzed using qRT-PCR (Figure 6). The expression of *miR156a, miR157a, miR159a, miR172, miR396*, and *miR826* in 2-day-old seedlings was all up-regulated significantly comparing with those in 1-day-old seedlings. Among these genes, *miR156a, miR157, miR159a, miR172*, and *miR826b* expression levels increased in 3-day-old seedlings and subsequently decreased in 5-day-old seedlings. In contrast, the amounts of *miR160, miR166, miR167, miR408*, and *miR826a* transcripts were decreased in 3-day-old seedlings and increased in 5-day-old seedlings. In 9-day-old seedlings, all the analyzed miRNAs were up-regulated comparing with those in 1-day-old seedlings except for *miR160a-5p* and *miR826b*.

### Prediction of *B. alboglabra* target genes

The roles of miRNAs in living organisms are associated with their sequence complementarity to specific mRNAs, ultimately leading to the inhibition of protein synthesis or cleavage of target mRNAs. Therefore, to determine the miRNA functions in cells, the miRNA targets need to be identified and characterized. To better understand the importance of miRNAs in processes occurring in *B. alboglabra* seedlings, we predicted their targets using the psRobot and TargetFinder programs. A total of 206 and 213 miRNA genes and 3167 and 3813 corresponding target genes were predicted in 2- and 9-day-old seedlings, respectively. Among these genes, 157 and 172 known miRNAs and 1305 and 1485 corresponding target genes were identified using psRobot in 2- and 9-day-old seedlings, respectively. Additionally, 182 and 193 known miRNAs and 2536 and 3113 corresponding target genes were detected with TargetFinder in 2- and 9-day-old seedlings, respectively.

**Figure 7:**
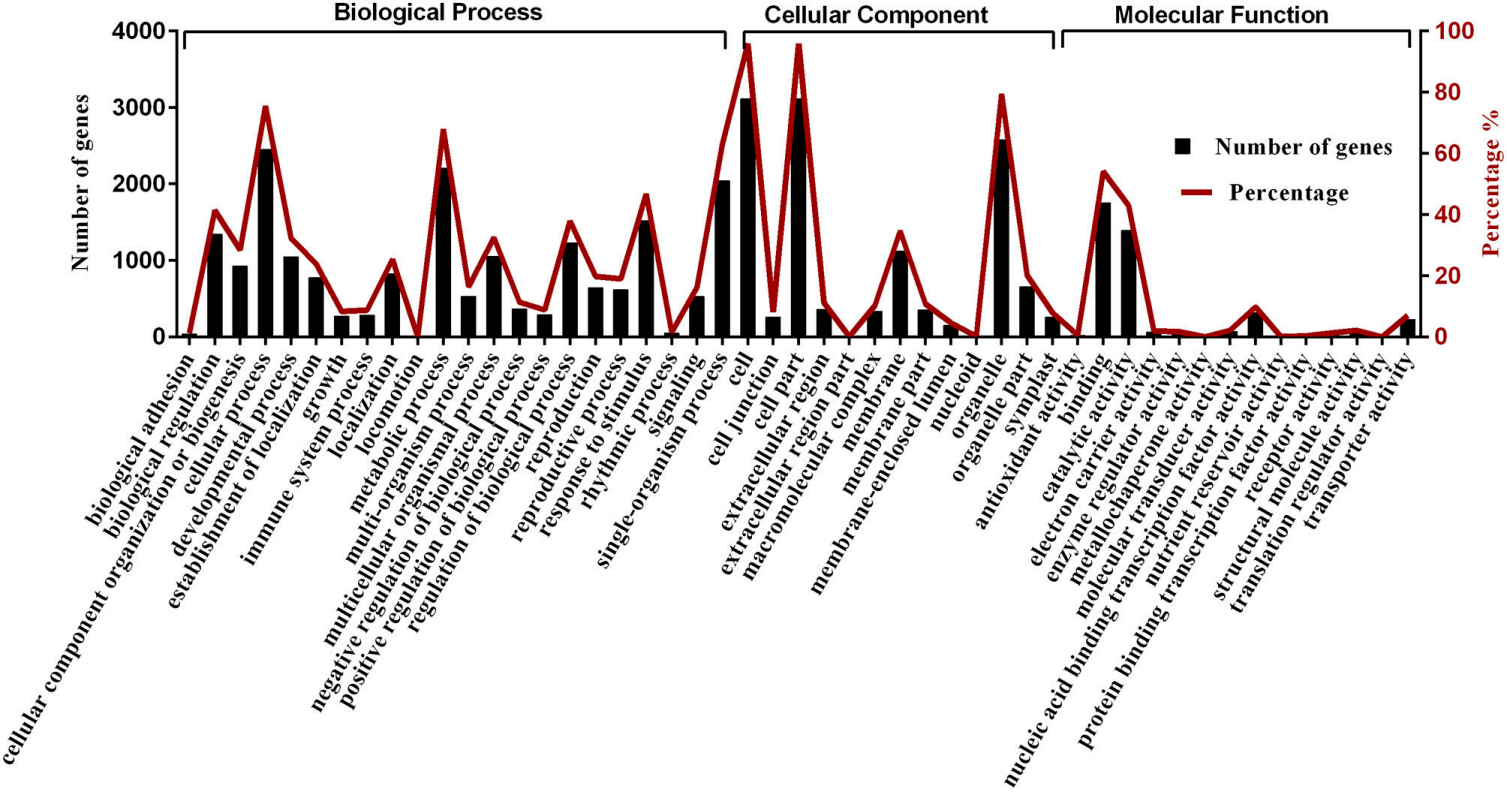
**GO (Gene ontology) annotation of predicted target genes of differentially expressed miRNAs in 2- and 9-day-old *B. alboglabra* seedling libraries**. Black column: target gene numbers of miRNAs in a specific category; Red line: percent of target gene numbers in a specific category. The X axis represented different categories of predicted target genes in *B. alboglabra*. The left Y axis is the target gene numbers of miRNAs in a specific category. The right Y axis is the percent of target gene numbers in a specific category.

We obtained gene ontology (GO) annotations from the Gene Ontology (http://www.geneontology.org) and NCBI (http://www.ncbi.nlm.nih.gov/COG, Gene ontology) databases. Gene annotations and classifications based on biological pathways (biological process: BP), cellular localizations (cellular component: CC), and molecular function (molecular function: MF) are provided in Figure 7. We classified 3243 differentially expressed genes into 22 BP, 13 CC, and 14 MF. The results showed that redundancy of gene functions existed in each category. Among the BP categories, “cellular process” contained the most genes (2448 genes), followed by “metabolic process” (2202 genes), and “single-organism process” (2042 genes). There were 1753 and 1392 genes in the “binding” and “catalytic activity” MF categories, respectively. After completing a GO enrichment analysis of the BP categories, we determined that “regulation of transcription” (GO: 0006355) contained the most genes (i.e., 279 differentially expressed genes out of 2281 BP genes were identified after cluster and genome frequency comparisons), followed by “formation of organ boundary” (GO: 0010160) and “RNA 5′-end processing” (GO: 0000966) (Supplementary Table S5).

### Expression of miRNAs targets during *B. alboglabra* seedling establishment

**Figure 8:**
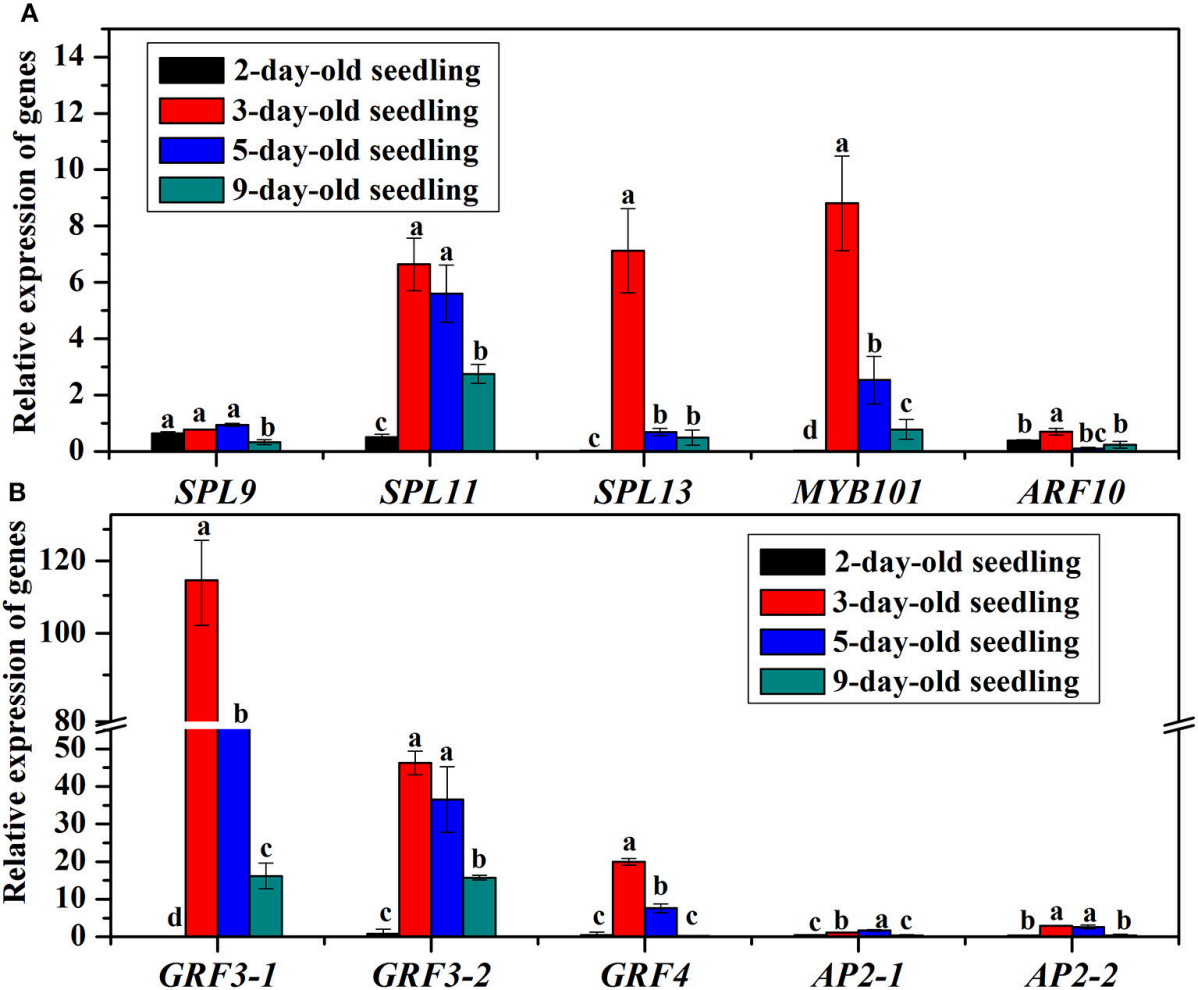
**(A)** Relative expression of target genes *SPL9, SPL11, SPL13, MYB101*, and *ARF10* in 2, 3, 5, and 9-day-old *B. alboglabra* seedlings. **(B)** Relative expression of target genes *GRF3-1, GRF3-2, GRF4, AP2-1*, and *AP2-2* in 2−, 3, 5, and 9-day-old *B. alboglabra* seedlings. The X axis represented target genes analyzed in *B. alboglabra*. The Y axis is the relative expression of target genes. Black column: relative expression of target genes in 2-day-old seedlings; Red column: relative expression of target genes in 3-day-old seedlings; Blue column: relative expression of target genes in 5-day-old seedlings; Indigo column: relative expression of target genes in 9-day-old seedlings. The expressions of target genes were calculated relative to those in 1-day-old seedlings. Error bars indicate the SE of three replicates. The differences labeled with different letters were considered significant at *p* < 0.05.

The *bal-SPL, bal-MYB101, bal-ARF10, bal-GRF*, and *bal-AP2* genes, which are the targets of *bal-miR156, bal-miR159, bal-miR160, bal-miR396*, and *bal-miR172*, respectively, expressed at various levels (Figure 8). The possible cleavage sites of target genes of corresponding miRNA were listed in Supplementary Table S6. The amounts of all analyzed target genes were less in 2-day-old seedlings than that in 1-day-old seedlings. The expression levels of *bal-SPL11, bal-SPL13, bal-MYB101*, and *bal-GRF3* were increased at day 3 then proceeded to decrease until day 9. Other genes like *bal-SPL9, bal-ARF10*, and *bal-AP2* were always at low expression levels during the seedling establishment stage.

## Discussion

### Size distribution changes of small RNAs during the establishment of *B. alboglabra* seedlings

The sequencing and analysis of Chinese kale sRNA libraries generated more than 10 million sequences. Similar to the sRNA length distribution of small RNAs observed in other plants (e.g., *Brassica napus*) (Zhao et al., 2012), the most abundant sRNAs with or without perfect genomic matches were 24 nt long in the two analyzed libraries. The next most abundant sRNAs were 21 nt long. Interestingly, we observed that the size distribution of Chinese kale sRNAs changed during the seedling-establishment stage. The 24-nt sRNAs accounted for 37.65 and 26.65% of the total sRNAs in 2- and 9-day-old seedlings, respectively. In contrast, the proportion of 21-nt sRNAs increased from 17.00% (2-day-old seedlings) to 26.47% (9-day-old seedlings) over time.

The sRNA sizes are likely influenced by the intrinsic structural characteristics of a Dicer-like (DCL) protein (Qi and Hannon, 2005). In higher plants, DCL3 functions in the heterochromatic pathway to produce 24-nt heterochromatic small interfering RNAs (siRNAs) and long miRNAs (Xie et al., 2004). In tomato plants, constitutive trans-activation of *SlDCL3* decreases *SlDCL3* levels by approximately 64%, resulting in considerably decreased 24-nt sRNA levels and a significant increase in the abundance of 21- and 22-nt sRNAs (Kravchik et al., 2014). Genetic and biochemical studies have revealed that *A. thaliana* DCL3 catalyzes the production of 24-nt siRNAs and miRNA variants (Xie et al., 2004; Vazquez et al., 2008). Post-transcriptional silencing of *DCL3* (i.e., RNA interference) in rice reduces the dominance of 24-nt sRNAs. Additionally, in *dcl3a-17* mutants, the 21-nt size class is overrepresented, while 24-nt sRNAs are underrepresented relative to the levels in wild-type plants (Wu et al., 2010). Consistent with the reduced levels of 24 nt sRNAs in 9-day-old seedlings, the expression level of *DCL3* was lower in 9-day-old seedlings than that in 2-day-old seedlings (Supplemental Figure S1) in our study.

Differences in sRNA lengths are closely related to plant development stage. In a previous study, more 24-nt sRNAs were generated from a *B. napus* mature seed library than from a developing seed library (Körbes et al., 2012). Huang et al. also reported that the abundance of 24-nt sRNAs increases during the early *B. napus* seed development stage (Huang et al., 2013). The 24-nt sRNAs mainly consist of siRNAs derived from repeats and transposable elements, which play key roles in loci silencing (Chen, 2010). The decrease in the 24-nt sRNA levels in 9-day-old seedlings indicates the repression of repeats and transposons varies during the seedling-establishment stage. The increased proportion of 21-nt sRNAs during the establishment of Chinese kale seedlings may also be functionally relevant.

### miRNA functions during the establishment of *B. alboglabra* seedlings

Auxin is essential for plant growth and development. Auxin response factors (ARFs), which are released by the auxin receptor E3 ubiquitin ligase complex (i.e., SCF^TIR1^), regulate the expression of a large set of auxin-responsive genes (Mockaitis and Estelle, 2008). *miR160* and its target genes, *ARF10, ARF16*, and *ARF17* are important for ensuring normal plant development. Plants expressing miR160-resistant *ARF genes* have been used to characterize the biological functions of *ARF10* (Liu et al., 2007), *ARF16* (Wang et al., 2005), and *ARF17* (Mallory et al., 2005). *ARF10* plays a key role during seed germination, while the repression of *ARF16* and *ARF17* is essential for the development of embryos, roots, leaves, and floral organs. The germinating seeds of *mARF10* plants (i.e., transgenic plants with silent mutations in *ARF10*) are hypersensitive to abscisic acid and overexpress abscisic acid -responsive genes. Additionally, the *mARF10* seedling cotyledons are abnormal (Liu et al., 2007). A recent study concluded that ARF10 and ARF16 activate ABI3 (abscisic acid insensitive 3) transcription during the inhibition of seed germination in *A. thaliana*. Additionally, *arf10arf16* double mutants exhibit a significantly higher seed germination rate than wild-type controls based on cotyledon greening (Liu et al., 2013). These results indicate that miR160 has a key function during seed germination. In this study, we observed that *miR160a-5p* expression was higher in 9-day-old seedlings than in 2-day-old seedlings, suggesting the importance of miR160 and the repression of auxin signaling during the Chinese kale seedling-establishment stage (i.e., period between imbibition and cotyledon expansion). However, *miR160a-3p* was down-regulated in 9-day-old seedlings. This may have been because that the negative regulation of *ARF10* by miR160 is through its 3′ regulatory sequence (Liu et al., 2007), which influences the establishment of Chinese kale seedlings.

The miR167-ARF6/8 regulatory module is critical for controlling cellular free- auxin levels, which affects the development of lateral root architecture in *A. thaliana* (Gutierrez et al., 2009) and soybean plants, as well as *A. thaliana* (Wu et al., 2006) and tomato (Liu et al., 2014) fertility. The expression of *miR167* in *Brassica* species exhibits a similar pattern to that of *A. thaliana*, soybean, and tomato plants. Additionally, *A. thaliana* plant exhibit a strong miR167 signal in the seed coat, and a gradient-like expression pattern in embryonic cotyledons (Ágyi and Havelda, 2013). In *B. napus, bna-miR167* is preferentially expressed in the cotyledons and seed coat (Huang et al., 2013). In our study, the expression of *miR167* was changed during the seedling establishment stage. Based on this observation and the fact that *miR167* is highly expressed in *A. thaliana* and *B. napus* embryonic cotyledons, we hypothesized that miR167 is functional during the development of embryonic and seedling cotyledons.

During the seedling-establishment stage, leaf growth, especially cotyledon expansion, is particularly noticeable. Studies on leaf growth in model plants have determined that miR396-GRF regulatory modules regulate leaf size and shape by spatially and temporally affecting cell division and maturation (Debernardi et al., 2012; Gupta and Nath, 2015).

miR166 is highly conserved among terrestrial plants, and down-regulates the expression of five members of the class III homeodomain-leucine zipper transcription factor gene family (Prigge et al., 2005). Ectopic over-expression of miR166 results in an enlarged shoot apical meristem and enhanced vascular development in *A. thaliana* plants (Williams et al., 2005). Inhibition of the MIR166 family in transgenic plants leads to pleiotropic effects, including increased indole-3-acetic acid content and decreased indole-3-acetic acid sensitivity, and the development of upward-facing, spoon-shaped cotyledons and abnormal rosette leaves (Jia et al., 2015). During the establishment of Chinese kale seedlings, *miR166* expression was also up-regulated, indicating its involvement in normal leaf development.

In addition to the development-related miRNAs, stress-related miRNA miR408 were also highly expressed during the seedling establishment stage. Several reports have indicated that miR408-SPL regulatory module is affected by various stresses, including mechanical stress (Lu et al., 2005), dehydration (Kantar et al., 2010), and copper exposure (Yamasaki et al., 2007; Lu et al., 2015). The level of miR408 expression also considerably influences growth and development. Plants with the *miR408-OX* regulatory module exhibit increased growth vigor, even at the seedling stage, and higher fresh weights. Decreasing *miR408* content in *A. thaliana* leads to impaired growth (Zhang and Li, 2013). Under standard plant growth conditions, *miR408* is constitutively expressed in *A. thaliana* plants, as well as in Chinese kale seedlings. These results suggest miR408 is conserved among plant species, and is active throughout plant development.

### Glucosinolate biosynthesis-related miRNA in *B. alboglabra*

In response to nitrogen deficiency in *A. thaliana* plants, miR826 may regulate the expression of its target gene, *AOP2*, which encodes a 2-oxoglutarate-dependent dioxygenase involved in glucosinolate biosynthesis (Liang et al., 2012). Glucosinolates, which are rich in nitrogen and sulfur, are plant secondary metabolites produced mainly in *Brassica* species. During the establishment of Chinese kale seedlings, the *bal-miR829a* expression level was higher in 2-, 3-, 5-, and 9-day-old seedlings than in 1-day-old seedlings. These findings imply that glucosinolate degradation may provide plants with a source of nitrogen and sulfur.

In conclusion, the development-associated miRNAs were highly-expressed during the establishment of *B. alboglabra* seedling, as were stress- and metabolism-related miRNAs. Combined with the low level of targets *SPL9* and *AP2*, it was concluded that *miR156-SPL9* and *miR172-AP* modules play key roles during the *B. alboglabra* seedling establishment stage.

## Author contributions

ZL, RG, and XC designed research; RG, XC, and ZH performed research and wrote the paper; ZL, RG, XC, YD, ZH, and XX analyzed data. ZL, XX, ZH, and YD participated in the sequence analysis and helped to modify the manuscript. All authors have read and approved the manuscript for publication.

### Conflict of interest statement

The authors declare that the research was conducted in the absence of any commercial or financial relationships that could be construed as a potential conflict of interest. The reviewer PH and handling Editor declared their shared affiliation, and the handling Editor states that the process nevertheless met the standards of a fair and objective review.
