# Supplementary material for: Identification of miRNAs Affecting the Establishment of Brassica Alboglabra Seedling
Source: Front Plant Sci. 2016 Nov 22;7:1760. doi: 10.3389/fpls.2016.01760 (PMC5147431; doi:10.3389/fpls.2016.01760)
Supplement: Supplementary file 8 [file DataSheet1.DOCX]

**Identification of MiRNAs Affecting the Establishment of *Brassica alboglabra* Seedling**

Rongfang Guo, Xu XuHan^*^, Zhongxiong Lai^*^

Correspondence:

Prof. Zhongxiong Lai

College of Horticulture

Fujian Agriculture and Forestry University

Shangxiadian Road 15,

Fuzhou 350002, China

[laizx01@163.com](mailto:laizx01@163.com)

Prof. Xu XuHan

Institut de la Recherche Interdisciplinaire de Toulouse

Toulouse

France

[xxuhan@163.com](mailto:xxuhan@163.com)

Supplemental Figure S1: The expression of *DCL3* in 2- and 9-day-old *B. alboglabra* seedlings.
